# Familial Alzheimer’s Disease Mutations in *PSEN1* Lead to Premature Human Stem Cell Neurogenesis

**DOI:** 10.1016/j.celrep.2020.108615

**Published:** 2021-01-12

**Authors:** Charles Arber, Christopher Lovejoy, Lachlan Harris, Nanet Willumsen, Argyro Alatza, Jackie M. Casey, Georgie Lines, Caoimhe Kerins, Anika K. Mueller, Henrik Zetterberg, John Hardy, Natalie S. Ryan, Nick C. Fox, Tammaryn Lashley, Selina Wray

**Affiliations:** 1 Department of Neurodegenerative Disease, UCL Queen Square Institute of Neurology, London, UK; 2 Neural Stem Cell Biology Laboratory, The Francis Crick Institute, London, UK; 3 Queen Square Brain Bank for Neurological Disorders, Department of Clinical and Movement Neuroscience, UCL Queen Square Institute of Neurology, London, UK; 4 Department of Psychiatry and Neurochemistry, Institute of Neuroscience and Physiology, the Sahlgrenska Academy at the University of Gothenburg, Mölndal, Sweden; 5 Clinical Neurochemistry Laboratory, Sahlgrenska University Hospital, Mölndal, Sweden; 6 UK Dementia Research Institute at UCL, London, UK

**Keywords:** Alzheimer’s disease, iPSC, organoid, neurogenesis, hippocampus, PSEN1, NOTCH, γ-secretase

## Abstract

Mutations in presenilin 1 (*PSEN1*) or presenilin 2 (*PSEN2*), the catalytic subunit of γ-secretase, cause familial Alzheimer’s disease (fAD). We hypothesized that mutations in *PSEN1* reduce Notch signaling and alter neurogenesis. Expression data from developmental and adult neurogenesis show relative enrichment of Notch and γ-secretase expression in stem cells, whereas expression of *APP* and β-secretase is enriched in neurons. We observe premature neurogenesis in fAD iPSCs harboring *PSEN1* mutations using two orthogonal systems: cortical differentiation in 2D and cerebral organoid generation in 3D. This is partly driven by reduced Notch signaling. We extend these studies to adult hippocampal neurogenesis in mutation-confirmed postmortem tissue. fAD cases show mutation-specific effects and a trend toward reduced abundance of newborn neurons, supporting a premature aging phenotype. Altogether, these results support altered neurogenesis as a result of fAD mutations and suggest that neural stem cell biology is affected in aging and disease.

## Highlights

• In neurogenesis, *PSEN1* expression is enriched in progenitors, as it is for *APP* in neurons
• Inhibiting β-secretase has little effect on neurogenesis, contrary to γ-secretase
• Familial Alzheimer’s disease mutations in *PSEN1* cause premature neurogenesis
• Trend toward fewer newborn neurons in familial AD postmortem hippocampi

## Graphical Abstract

**Figure:**
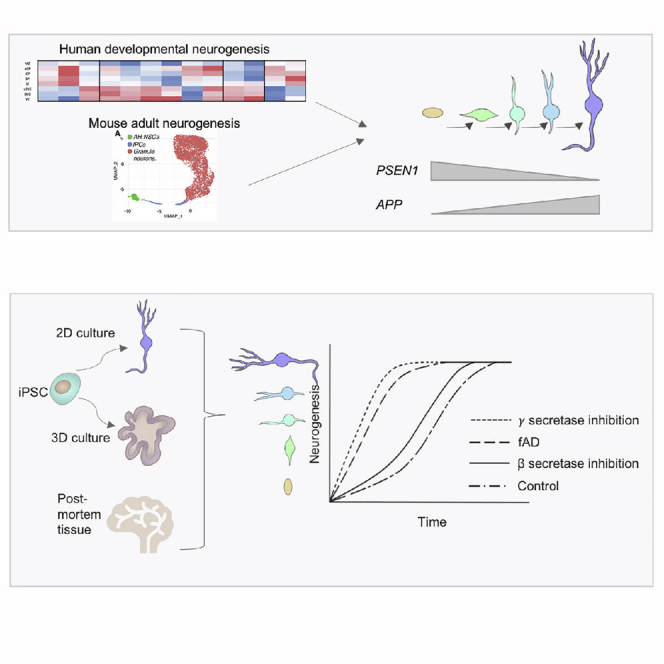

## Teaser

Arber et al. employ human iPSC neurogenesis to model adult hippocampal neurogenesis, investigating familial Alzheimer’s disease (fAD) mutations. In contrast to *APP*, *PSEN1* and γ-secretase components are enriched in neural progenitors and mutations drive premature neurogenesis. Postmortem fAD hippocampi show corresponding trends toward altered neurogenesis.

## Introduction

Mutations in amyloid precursor protein (*APP*) and presenilin 1 and 2 (*PSEN1/2*) cause familial Alzheimer’s disease (fAD) (Goate et al., 1991; Levy-Lahad et al., 1995; Sherrington et al., 1995). PSEN1/2 and APP exist on the same molecular pathway: PSEN1 is the catalytic subunit of γ-secretase, a transmembrane enzyme that cleaves APP to generate β-amyloid (Aβ). The amyloid cascade hypothesis theorizes that fAD mutations increase either the amount of Aβ produced or the proportion of amyloidogenic Aβ moieties, leading to aggregation and predisposing neurodegeneration (Selkoe and Hardy, 2016). *PSEN1* mutations achieve this via destabilization of the enzyme-substrate interaction, increasing the relative levels of longer, more aggregation-prone forms of Aβ (Chávez-Gutiérrez et al., 2012; Szaruga et al., 2017).

In addition to APP, γ-secretase cleaves a host of other type I transmembrane substrates (reviewed in Haapasalo and Kovacs, 2011), altered cleavage of which could potentially contribute to the clinical heterogeneity seen among *PSEN1* mutations (Ryan et al., 2016). One example is the cell-signaling factor Notch. After binding of the Notch receptor to Jagged and DLL ligands on neighboring cells, α- and γ-secretases cleave the Notch protein to release a transcriptionally active intracellular domain (Notch intracellular domain [NICD]). A subset of fAD mutations has been shown to reduce the cleavage of Notch by γ-secretase (Chávez-Gutiérrez et al., 2012; Song et al., 1999), thereby decreasing Notch signaling.

Notch signaling is required for several cell-contact-dependent processes, an example of which is the maintenance of stemness in the stem cell niche. As such, human stem cell neurogenesis *in vitro* relies on Notch signaling, and inhibition of Notch cleavage through γ-secretase inhibitors leads to rapid terminal differentiation of neural precursors (Borghese et al., 2010; Main et al., 2013; Woo et al., 2009). In addition, active Notch signaling is required for progressive rounds of differentiation from cortical progenitors, orchestrating temporal patterning toward different cortical-layer fates (Edri et al., 2015). Continued Notch signaling may be important for the persistence of stemness and the establishment of adult neurogenic cells (Edri et al., 2015), and there is evidence that tissue-resident neural stem cells (NSCs) maintain developmental neurogenesis programs in the adult brain (Berg et al., 2019).

Adult neurogenesis is the process whereby new neurons are generated from NSCs in the subventricular zone (SVZ) of the cortex and the dentate gyrus of the hippocampus (Altman and Das, 1965). It is agreed that adult neurogenesis is a rare event that drops sharply after birth, yet there is some contention over its persistence into old age (Boldrini et al., 2018; Sorrells et al., 2018). Decreased neurogenesis correlates with cognitive decline in Alzheimer’s disease (AD) patients when compared with non-demented individuals with Alzheimer’s-like pathology (Briley et al., 2016). Indeed, both *psen1* knockout mice (Bonds et al., 2015; Handler et al., 2000; Saura et al., 2004) and animals harboring fAD mutations (Hamilton et al., 2010; Haughey et al., 2002; Wen et al., 2002, 2004) show disrupted neurogenesis and reduced learning and memory. Enhanced neurogenesis has also been described in *psen1/2* double-knockout mice (Chen et al., 2008). In addition, rodent models of human APOE4 (Adeosun et al., 2014; Li et al., 2009) or apoE deficiency display enhanced neurogenesis (Li et al., 2009; Yang et al., 2011). However, effects may be nuanced, with increased neurogenesis in young mice and a subsequent reduction in neurogenesis in older apoE knockout mice (Tensaouti et al., 2018; Yang et al., 2011) and APOE4 mice (Tensaouti et al., 2018). In agreement, studies using human postmortem brain tissue showed that neurogenesis was reduced in AD patients (Moreno-Jiménez et al., 2019) and in early-onset AD patients (Crews et al., 2010) and was correlated with cognitive performance (Tobin et al., 2019). However, there is evidence to contradict these findings (Boekhoorn et al., 2006; Jin et al., 2004). Altogether, these data suggest that fAD mutations that perturb Notch signaling could negatively affect appropriate γ-secretase-dependent neurogenesis.

*PSEN1* has been shown to be critical for appropriate embryonic neurogenesis (Handler et al., 2000), which led us to investigate the effect of mutations in *APP* and *PSEN1* on stem cell neurogenesis using both induced pluripotent stem cell (iPSC) differentiation models and postmortem brain tissue from fAD mutation carriers. We hypothesized that mutations in *PSEN1* that reduce γ-secretase activity would lead to premature terminal differentiation via reduced Notch signaling, an effect not predicted for *APP* mutant cells.

## Results

### Spatiotemporal Restriction of Notch, γ-Secretase, and APP in Human Development

**Figure 1:**
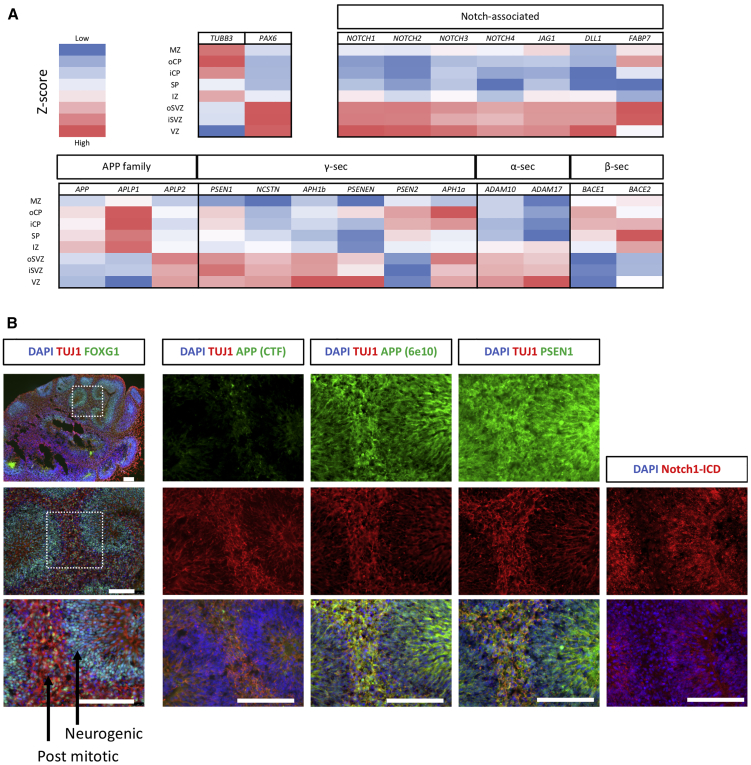
Notch, APP, and Their Cleavage Enzymes Are Spatiotemporally Restricted in Human Neurogenesis (A) Expression pattern across the cortical layers of human tissue between 15 and 21 weeks after conception using data from BrainSpan (Miller et al., 2014). *TUBB3* and *PAX6* are controls for neurons and precursors, respectively. Notch receptors (*NOTCH1*–*NOTCH4*), ligands (*JAG1* and *DLL1*), and the Notch readout gene *FABP7* (alias *BLBP*) are enriched in the progenitor layers. α-secretase displays enrichment in the progenitor layers, as does γ-secretase, which shows subunit-specific expression patterns. *APP* and β-secretase enzymes are enriched in neuronal layers. (B) Serial sections and immunocytochemical staining from a representative iPSC-derived cerebral organoid at 45 days post induction. FOXG1 staining confirms forebrain identity. APP is enriched in neuronal regions (marked by TUJ1) and cleaved; active Notch is found in proliferative regions. PSEN1 is more evenly distributed. Scale bar represents 100 μm. γ-, β-, and α-sec, γ-, β-, and α-secretase, respectively; MZ, marginal zone; oCP and iCP, outer and inner cortical plate, respectively; SP, subplate; IZ, intermediate zone; iSVZ and oSVZ, inner and outer subventricular zone respectively; VZ, ventricular zone.

To compare γ-secretase-, Notch-, and APP-associated gene expression during human development, data were leveraged from the BrainSpan reference atlas (Miller et al., 2014). Cortical-layer-specific gene expression relative to the overall mean was compared using expression data from fetal tissue 15 to 21 weeks after conception (Figure 1A).

As expected, Notch ligands and receptors are enriched in the ventricular zone and the SVZ of the developing cortex, representing progenitor cell populations. In general, γ-secretase subunits displayed similar enrichment in the progenitor layers with alternate subunits displaying differential distributions; for example, *PSEN1* and *APH1B* displayed enrichment in proliferative cells, whereas *PSEN2* and *APH1A* showed enrichment in terminally differentiated neurons. In addition, α-secretase expression was enriched in the progenitor populations, whereas β-secretase and *APP* expression was highest in postmitotic neuronal layers. In addition, APP family members showed differing expression patterns, with *APLP2* enrichment in progenitors in contrast to the neuronally expressed *APP* and *APLP1*.

To validate these expression data, we investigated protein distribution in developing human cerebral organoids via immunofluorescence (Figure 1B; Figure S1). Cells follow intrinsic differentiation cues to organize into a proliferative zone, akin to the SVZ of the developing brain, and a neuronal cortical plate-like region as neurons mature and migrate from the SVZ (Renner et al., 2017). Active Notch signaling was highest in neurogenic regions, shown via detection of the cleaved NICD. In contrast, APP was restricted to postmitotic neuronal regions. PSEN1 displayed a more ubiquitous expression pattern.

These data support a spatiotemporal separation of the γ-secretase substrates APP and Notch in different developmental states. β- and α-secretase expression also show distinction, a finding supported by data showing that APP fragments generated by α-secretase (Aβ16) appear early in iPSC differentiation compared with the later β-secretase-dependent fragments (Aβ40 and Aβ42) (Bergström et al., 2016). In addition, the data support distinction of alternate γ-secretase subunits between cell identities, building on reports that PSEN1 and PSEN2 exhibit differences in subcellular compartmentalization (Sannerud et al., 2016).

### γ-Secretase and β-Secretase Inhibition Have Distinct Effects on Neurogenesis

Given the spatiotemporal distinction between γ- and β-secretase subunit expression, we next investigated the functional impact of inhibiting both enzymes on stem cell neurogenesis.

**Figure 2:**
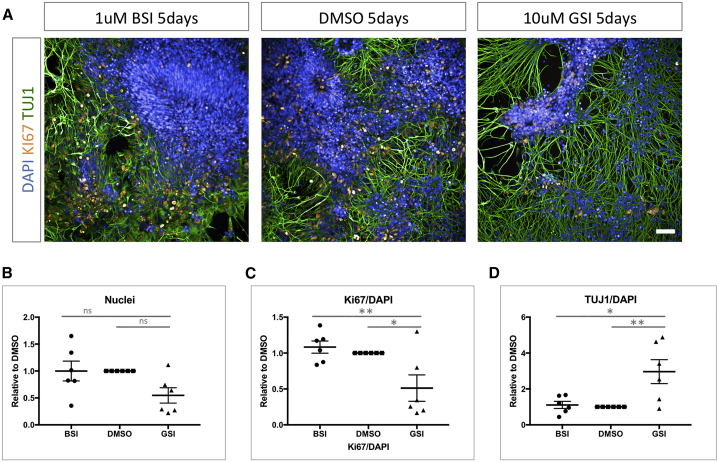
β-Secretase and γ-Secretase Inhibition Differentially Affect Neurogenesis (A) Representative images of day 27 iPSC-derived neural precursors treated with β-secretase inhibitor (BSI; LY2886721, 1 μM), vehicle (DMSO), and γ-secretase inhibitor (GSI; DAPT, 10 μM). Ki67 marks dividing cells, and TUJ1 depicts terminally differentiated neurons. (B–D) Quantification of 6 independent inductions and the effect of β- and γ-secretase inhibition on total cell number, dividing cells (Ki67), and TUJ1 postmitotic neurons via high-content imaging. ^∗^p < 0.05, ^∗∗^p < 0.01 via ANOVA with post hoc Tukey’s analysis, as indicated in Table S1. Scale bar represents 50 μm. Error bars represent standard error of the mean.

As previously described in the literature, DAPT-mediated γ-secretase inhibition of neural precursors led to a dramatic exit from the cell cycle concurrent with terminal differentiation (Figure 2; Figure S2) (Borghese et al., 2010; Main et al., 2013; Woo et al., 2009). This is demonstrated via reduced total cell numbers, a lower population of cells expressing the proliferative marker Ki67, and increased expression of the neuronal marker TUJ1. In contrast, treatment with the β-secretase inhibitor LY2886721 led to no significant effect on the balance between proliferation and neurogenesis. This finding supports the distinct roles of β- and γ-secretases on neural development.

### fAD Mutations Cause Premature Differentiation *In Vitro*

We next investigated the consequence of mutations in *PSEN1* and *APP* on neurogenesis using a panel of 5 control iPSC lines and 7 lines harboring different fAD mutations at day 27 of cortical differentiation, a time point of neuronal commitment (Shi et al., 2012). The panel consisted of two independent lines with the *APP* V717I mutation and five separate *PSEN1* mutations: intron 4 deletion (int4del), Y115H, M139V, M146I, and R278I (see STAR Methods) (previously described in Arber et al., 2020).

**Figure 3:**
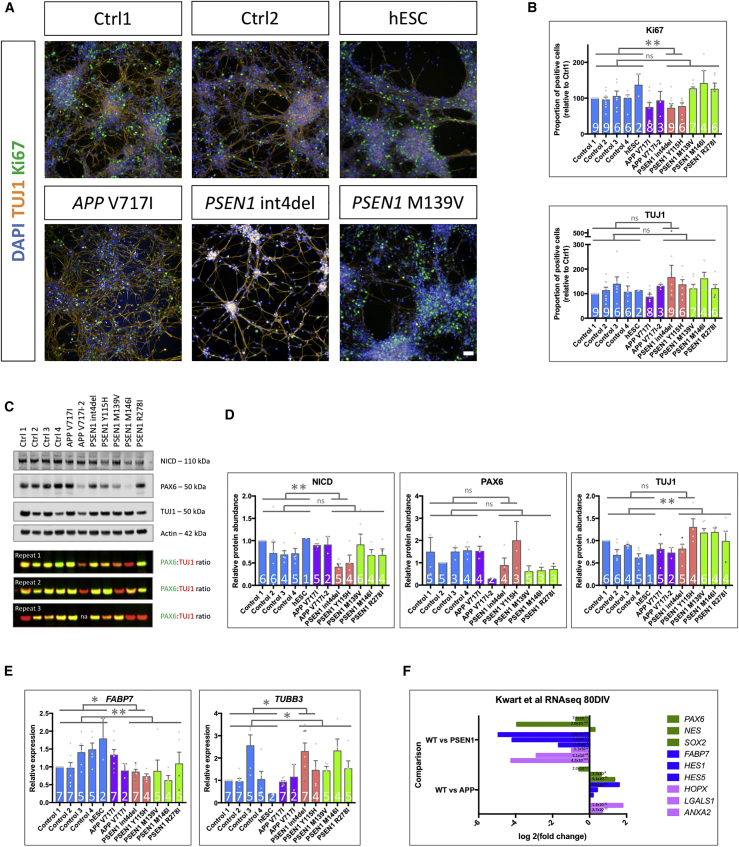
Mutations in *PSEN1* Reduce Notch Signaling and Lead to Premature Terminal Differentiation (A) Representative images of day 27 iPSC-derived neural progenitors labeled for dividing cells (Ki67) and postmitotic neurons (TUJ1). (B) High-content analysis and quantification of the relative proportion of proliferative (Ki67 positive) and neuronal (TUJ1 positive) cells. (C and D) Representative western blot and quantification for the cleaved, active Notch intracellular domain (NICD), PAX6 neural progenitor marker, and TUJ1 neuronal marker. Note, due to variability in Ctrl1, PAX6 data were normalized to Ctrl2. Due to similar molecular weights, the ratio of PAX6 to TUJ1 intensity represents a measure of terminal differentiation. na, not available. (E) qPCR analysis of the expression of the Notch readout gene *FABP7* (alias *BLBP*) and *TUBB3* (neuronal tubulin). (F) RNA sequencing (RNA-seq) expression data from Kwart et al. (2019), demonstrating expression of neural stem cell (NSC) markers (*PAX6*, *NES*, and *SOX2*), Notch signaling readout genes (*FABP7*, *HES1*, and *HES5*), and adult NSC markers (*HOPX*, *LGALS1*, and *ANXA2*) (Berg et al., 2019; Edri et al., 2015) in 80 days *in vitro* (DIV) iPSC-derived neurons from wild type (WT) compared with isogenic *PSEN1* or *APP* mutant lines. Adjusted p values are represented within the histogram. Blue, control; purple, APP mutant cells; pink, mutations in the PSEN1 extracellular loop; green, mutations in PSEN1 transmembrane and intracellular domains. The number of independent neural inductions is shown within the histograms. Data from different iPSC clones are depicted by gray data points (*APP* V717I, *PSEN1* int4del, and *PSEN1* R278I). ^∗^p < 0.05, ^∗∗^p < 0.01, ^∗∗∗^p < 0.001 via ANOVA with post hoc Tukey’s analysis (normal distribution tested via the Shapiro-Wilk test), as indicated in Table S1. Scale bar represents 50 μm. Error bars represent standard error of the mean.

Morphologically, the two lines harboring the *PSEN1* mutations int4del and Y115H displayed a striking premature differentiation phenotype, shown via earlier formation of neural projections relative to other lines (Figure 3A; Figure S2). We have previously shown that these mutations, which affect the extracellular substrate docking domain of PSEN1 (Somavarapu and Kepp, 2016; Takagi-Niidome et al., 2015), potentially reduce γ-secretase activity to a larger degree than the other mutations tested (Arber et al., 2020). Therefore, subgroup analysis of these lines was performed (pink histograms). High-content imaging, qPCR expression data, and western blot analysis supported a small but consistent premature differentiation phenotype in all lines harboring *PSEN1* mutations (int4del, Y115H, M139V, M146I, and R278I) (Figures 3A–3E). High-content imaging was used to assess levels of the postmitotic neuronal marker TUJ1 and a marker of proliferating precursor cells, Ki67 (Figures 3A and 3B). When compared with pooled control data, the *PSEN1* int4del and Y115H lines showed significantly reduced numbers of proliferative progenitor cells (p = 0.0081). Furthermore, when all lines bearing *PSEN1* mutations were pooled and compared with pooled control data, there was a trend toward an increased proportion of postmitotic neurons. Western blotting revealed a reduction in levels of cleaved, active Notch in lines bearing *PSEN1* mutations (Figures 3C and 3D), representing reduced γ-secretase-dependent Notch signaling, a reduction that was especially striking in the *PSEN1* int4del and Y115H lines (p = 0.0047). This was in addition to a trend toward decreased levels of neural progenitor marker PAX6 and increased levels of TUJ1 (p = 0.0028) (Figures 3C and 3D). qPCR analysis was then used to assess the expression levels of the brain-specific Notch signaling readout gene *FABP7* (alias *BLBP*) (Anthony et al., 2005), which displayed significantly reduced (p = 0.0083) expression in lines harboring *PSEN1* mutations compared with controls (Figure 3E). This was accompanied by significantly increased (p = 0.0310) expression of the neuronal-specific *TUBB3* gene (for which TUJ1 is the gene product). Progenitors harboring the *APP* V717I mutation displayed differentiation comparable to that of controls. *FABP7* expression was 20–40 times higher than that of the HES family genes *HES1*, *HES5*, and *HEY1*, alternate Notch readout genes that showed largely a similar expression pattern among genotypes.

The increased expression of neuronal markers and reduced abundance of progenitor cells in cultures harboring *PSEN1* mutations can be depicted by the ratio of PAX6 to TUJ1 protein abundance, made possible because of similar molecular weights (Figure 3C). This finding supports premature neuronal commitment. A depletion of NSC marker expression in *PSEN1* mutant neuronal cultures compared with isogenic control cultures can be independently verified in published expression data (Figure 3F) (Kwart et al., 2019).

**Figure 4:**
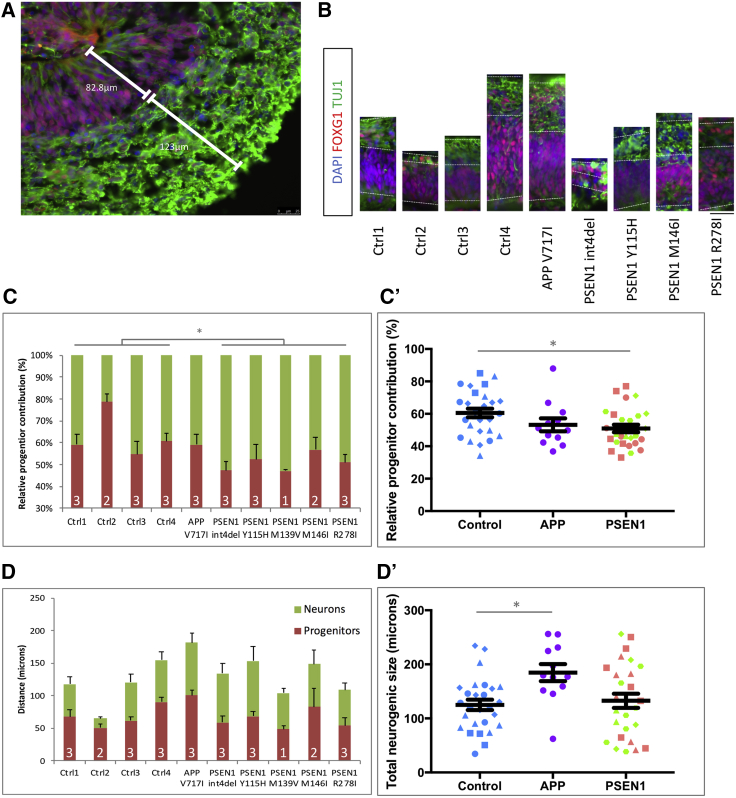
iPSC-Derived Cerebral Organoids Exhibit Premature Neurogenesis in *PSEN1* Mutant Lines (A and B) Depictions of the relative contribution of proliferative progenitors (FOXG1+/TUJ1−) and committed neurons (TUJ1+) within the neurogenic niches of iPSC-derived cerebral organoids. Quantifications were made from the basement membrane to the neural boundary (in micrometers) in at least two neurogenic regions per organoid. (B) is to scale, and the scale bar represents 25 μm. (C) Quantification of the relative contribution of the neural progenitors and neurons for individual control, *APP*, and *PSEN1* mutation lines. (C′) shows the same data grouped by genotype. (D) Quantification of the overall size of the neurogenic regions for each line (progenitor contribution plus neural contribution). (D′) shows the same data grouped by genotype. The number of independent organoid batches is shown within the histograms. ^∗^p < 0.05, ^∗∗^p < 0.01, ^∗∗∗^p < 0.001 via ANOVA with post hoc Tukey’s analysis, as indicated in Table S1. Ctrl1, blue circles; Ctrl2, blue squares; Ctrl3, blue triangles; Ctrl4, blue diamonds; *APP* V717I, purple circles; *PSEN1* int4del, pink circles; *PSEN1* Y115H, pink squares; *PSEN1* M139V, green triangles; *PSEN1* M146I, green diamonds; *PSEN1* R278I, green hexagons. Error bars represent standard error of the mean.

To validate the observed early terminal differentiation, we employed an orthogonal iPSC neurogenesis model, namely, 3D cerebral organoid differentiation. The relative contribution of progenitor cells versus committed neurons was assessed by immunocytochemistry (Figures 4A and 4B). This approach supported an increased neuronal contribution in *PSEN1* mutant lines relative to neural progenitors (p = 0.0266) (Figure 4C). In contrast, *APP* V717I lines displayed significantly larger total neurogenic regions (p = 0.0133), albeit with relative precursor and neuronal contributions similar to those of controls (Figure 4D).

These data support a premature differentiation phenotype in iPSC lines harboring mutations in *PSEN1*, and this effect can partly be explained via reduced Notch signaling. Analysis of two iPSC lines harboring the *APP* V717I mutation suggest premature differentiation may be specific to *PSEN1* mutant lines. The APOE genotype has been reported to affect neurogenesis (Adeosun et al., 2014; Li et al., 2009), but regrouping our data based on the APOE genotype of each line did not explain this phenotype, suggesting that *PSEN1* mutations are indeed driving this effect (Figure S3).

### Expression of Notch-, App-, and β/γ-Secretase-Associated Genes in Mouse Adult Hippocampal Neurogenesis

**Figure 5:**
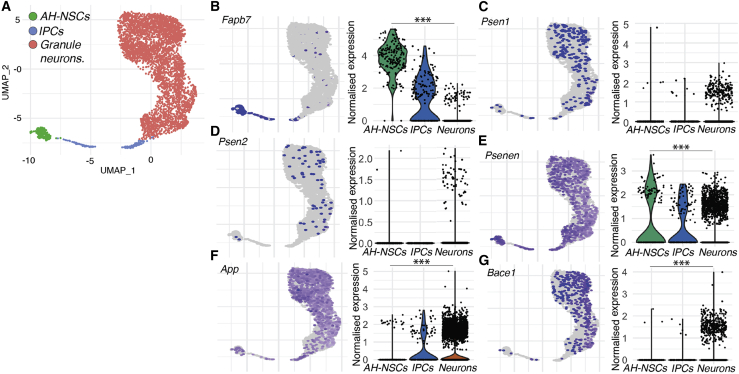
Expression of Notch Signaling Components and AD-Related Genes in the Mouse Adult Hippocampal Neurogenic Lineage (A) Low-dimensional representation of transcriptional space (UMAP plot), showing single-cell transcriptomes of adult hippocampal neural stem cells (AH-NSCs, green), intermediate neuronal progenitor cells (IPCs, blue), and granule neurons (red) derived from the hippocampus of postnatal mice from a publicly available dataset (Hochgerner et al., 2018). (B) Expression of the Notch signaling target gene *Fabp7* is higher in NSCs than in neurons. (C–E) γ-secretase genes *Psen1* and *Psen2* are expressed in all cell types but below the threshold for differential gene expression analyses. The *Psenen* subunit of γ-secretase is significantly higher in NSCs than in neurons. (F) Expression of *App* is enriched in neurons compared with NSCs. (G) Expression of the β-secretase gene *Bace1* is enriched in neurons. Wilcoxon rank-sum test, with the genome-wide false discovery rate (FDR)-corrected p value in (B)–(G). ^∗∗∗^p < 0.001, n.s., p > 0.05.

Previously reported single-cell RNA sequencing (scRNA-seq) data delineating neurogenic lineages from mouse hippocampi (Hochgerner et al., 2018) was reanalyzed to explore the expression of Notch, App, and γ-secretase components (Figure 5). Relative expression enrichment was compared among NSCs, intermediate neural progenitor cells (IPCs), and hippocampal granule neurons (Figures 5B–5G).

The Notch signaling readout gene (*Fabp7*) was enriched in NSC populations compared with neurons (Figure 5B). γ-secretase components *Psen1* and *Psen2* were detected but below the threshold for differential gene expression analysis; however, the obligate γ-secretase subunit *Psenen* showed significant enrichment in NSCs (Figures 5C–5E). In contrast, *App* and *Bace1* expression displayed significant enrichment in neuronal cells compared with NSCs (Figures 5F and 5G).

These data resemble the findings from developmental neurogenesis expression patterns (Figure 1), reinforcing the hypothesis that developmental neurogenesis programs are maintained during adult neurogenesis despite differences in stem cell states (Berg et al., 2019; Hochgerner et al., 2018). These data also reinforce the role for γ-secretase and Notch signaling in NSCs *in vivo*.

### Neurogenesis in Postmortem fAD Tissue

We postulated that mutations in *PSEN1*, but not *APP*, might also affect adult hippocampal neurogenesis in the dentate gyrus of fAD patients. To examine the impact of fAD mutations on NSCs *in vivo*, hippocampi from mutation-confirmed fAD postmortem brains were compared with non-age-matched control brains (see STAR Methods). Manual counting analysis was conducted by three independent and blinded scorers. NSCs were quantified using the cytoskeletal marker NESTIN, and newly committed neurons were quantified via TUJ1-positive neurites crossing the granule cell layer of the dentate gyrus. TUJ1 is shown to be highly enriched in newly formed neurons, and expression reduces with cellular maturity (von Bohlen Und Halbach, 2007; Gómez et al., 2017). Thus, this measure provided a proxy for neurogenesis over a large temporal window, and cytoskeletal markers potentially may be less prone to the effects of postmortem delay that is seen for extracellular markers such as DCX (Boekhoorn et al., 2006) and PSA-NCAM.

**Figure 6:**
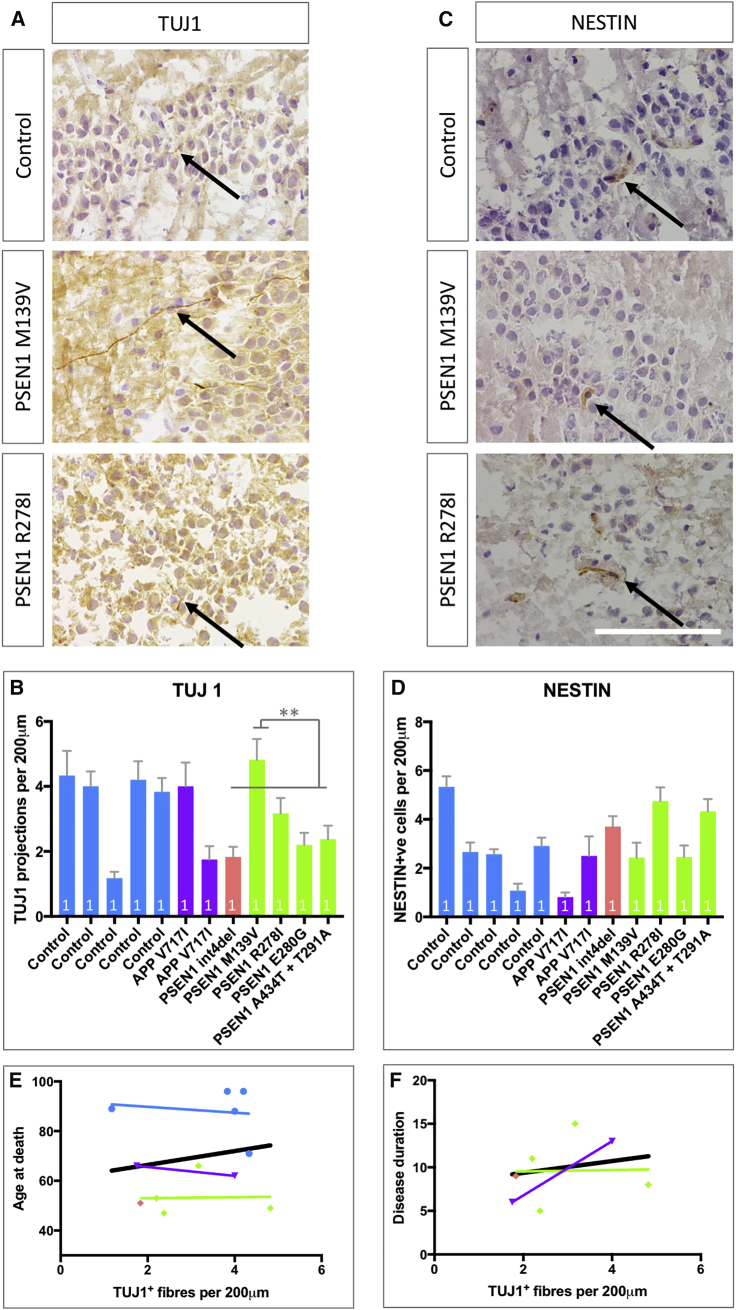
Analysis of Neurogenesis in Mutation-Confirmed fAD Postmortem Hippocampal Tissue (A) TUJ1-positive projections (black arrows) crossing the granule layer of the dentate gyrus in the hippocampus, employed as a marker for newly generated neurons. (B) Quantification of newborn neurons in the hippocampi per 200 μm. (C) NESTIN was used as a marker for NSCs. (D) Quantification of the number of NESTIN-positive cells in contact with the granule layer of the dentate gyrus per 200 μm. (E) Correlation of TUJ1 fibers (newborn neurons) with age at death per case. The black line is the regression line for all data. (F) Correlation of TUJ1 fibers (newborn neurons) with disease duration. The black line is the regression line for all data. Scale bar represents 100 μm. ^∗∗^p < 0.01 via ANOVA with post hoc Tukey’s analysis, as indicated in Table S1. R-squared values are shown in Table S1. Comparisons represent multiple fields of view for each brain (n = 1). Blue, control; purple, *APP* V717I; pink, mutations in the *PSEN1* extracellular loop; green, mutations in *PSEN1* transmembrane and intracellular domains. Error bars represent standard error of the mean.

Analyses showed that TUJ1-positive projections were detectable in all hippocampi (Figures 6A and 6B). There were no significant differences in the abundance of TUJ1-positive cells among genotype groups (Figure S4), despite clear differences among individuals. One control individual displayed few TUJ1-positive fibers, and this data point may skew otherwise significant differences, namely, reduced neurogenesis in fAD cases. Despite the lack of significance, interesting mutation-specific comparisons exist, including a significant increase in the number of TUJ1-positive neurites in the *PSEN1* M139V case compared with the other *PSEN1* mutations (comparisons made using multiple fields of view from individual cases). The *PSEN1* M139V mutation has been shown to have little effect on Notch endoproteolytic cleavage (Chávez-Gutiérrez et al., 2012). The *in vitro* data in this study are consistent with a less pronounced phenotype in the *PSEN1* M139V iPSC line compared with other fAD mutations, e.g., NICD western blotting (Figures 3C and 3D).

Next, we reasoned that premature differentiation observed *in vitro* may correspond to a depletion of the stem cell pool *in vivo*, similar to the effects of aging (Encinas et al., 2011). Quantification of NESTIN-positive NSCs in the granule cell layer highlighted consistent levels of NESTIN staining across all genotypes, arguing against an effect on the stem cell pool in fAD (Figures 6C and 6D; Figure S4).

When neurogenesis (TUJ1-positive neurite density) was correlated with age at death in control cases (Figure 6E), a trend toward reduced neurogenesis with advancing age was observed, supporting an overall reduction in neurogenesis with increasing age. The data highlight the younger age at death of the fAD cases but also a reduced age effect in the *PSEN1* mutation carriers, shown by the gradient of the regression. This suggests that the effect of *PSEN1* mutations on neurogenesis is dominant over age. In addition, we observed a trend for increased neurogenesis in individuals with longer disease duration (Figure 6F).

It is crucial to acknowledge the rarity of fAD postmortem tissue availability and the low number of brains analyzed in this study. However, interesting trends emerge that require further investigation. Neurogenesis has been shown to decline with age (Ben Abdallah et al., 2010; Leuner et al., 2007; Spalding et al., 2013). The lack of significant difference between fAD hippocampi (average age at death of 56 years; see STAR Methods) and control hippocampi (average age at death of 88 years) supports a dominant effect of the mutations on neurogenesis, in addition to the effect of aging.

## Discussion

This study demonstrates that the process of neurogenesis is affected by mutations that cause fAD. Mutations in *PSEN1* lead to a small yet significant premature differentiation phenotype, whereas the V717I mutation in *APP* may increase total neurogenesis. The *PSEN1* mutations int4del and Y115H in the substrate docking domain of the enzyme (Somavarapu and Kepp, 2016; Takagi-Niidome et al., 2015) have the strongest phenotype.

These data support previous observations in the literature. The *PSEN1* S169del and A246E mutations have been shown to lead to premature iPSC neurogenesis (Yang et al., 2017). The authors propose that this phenotype could be driven by changes to Notch signaling but also suggest defects in the Wnt signaling pathway. Similar findings of premature differentiation have been described for sporadic AD-patient-derived iPSC differentiation, whereby the authors describe changes in the REST-mediated transcriptional programs causing early differentiation and hyper-excitability in young neurons (Meyer et al., 2019).

Our data support the finding that dominantly inherited fAD mutations in *PSEN1* are sufficient to drive premature neural commitment. Sporadic AD iPSCs and *APOE* ε*4*-carrying cells can independently display premature neuronal commitment (Meyer et al., 2019), which suggests that this complex phenotype is likely to rely on multiple factors. That stated, the *APOE* genotype was not able to explain the presented data, because grouping the lines by *APOE* status did not highlight significant effects on neurogenesis (Figure S3).

Our data stratify fAD mutations as having different effects on neurogenesis. Mutations in the substrate docking domain of PSEN1 have the strongest premature neurogenesis phenotype compared with other PSEN1 mutation lines. What is more, *PSEN1* and *APP* mutations affect neurogenesis distinctly. These differences are presumed to relate to mutation effects on enzyme versus substrate, whereby *PSEN1* mutations alter γ-secretase-substrate interactions, including both Notch and APP (Szaruga et al., 2017), whereas *APP* mutations alter γ-secretase-APP interactions without affecting Notch. However, extracellular fragments of APP have been suggested to function as mitogenic signals in the adult neurogenic niche (Caillé et al., 2004), and conversely, the APP intracellular domain has been suggested to be part of a transcriptional complex that negatively regulates neurogenesis (Ma et al., 2008); therefore, *APP* mutations may affect neurogenesis through complex mechanisms. The enlarged neurogenic contribution of our *APP* mutation carrier organoids supports these studies.

We hypothesize that the observed neurogenesis effects in *PSEN1* mutants are mediated via changes to Notch signaling. Notch is processed in a manner similar to APP, whereby γ-secretase is responsible for the rate-limiting cleavage step, and both APP and Notch physically interact (Oh et al., 2005). Appropriate Notch signaling is required for most stem cell populations, and the Notch readout gene *FABP7* investigated in the present study can be used as a stem cell marker in the adult brain (Varela-Nallar et al., 2010). The importance for Notch-γ-secretase interactions is highlighted by the halting of γ-secretase inhibitor trials due to off-target effects on Notch signaling (Doody et al., 2013). In support of the role of aberrant Notch signaling in AD, Notch proteins have been shown to be a component of amyloid plaques in postmortem tissue (Brai et al., 2016) and the Notch ligand Jagged is reduced in AD (Marathe et al., 2017). Further work is required to disentangle cell-autonomous versus non-cell-autonomous pathomechanisms of fAD mutations on neurogenesis. The current work suggests a cell-autonomous reduction in Notch signaling as a major driver, with our recent work using an isogenic *PSEN1* int4del allelic series suggesting that this is not via a simple loss-of-function mechanism (Arber et al., 2019). However, previous work has implicated non-cell-autonomous functions of APP cleavage (Caillé et al., 2004), and the roles of secreted APP, distinct Aβ moieties, and Notch ligands should be further explored with respect to neurogenesis and fAD. With this in mind, care should be taken when comparing *psen1* knockout mice with fAD models; Meyer et al. (2019) did not witness altered Notch signaling in their iPSC models of sporadic Alzheimer's disease.

It is compelling that Notch pathway gene expression and γ-secretase components are spatiotemporally enriched in neural precursors. The enrichment for *APP* and β-secretases in postmitotic neurons reinforces the lack of effect of *APP* V717I on Notch signaling in neural progenitors. The different roles of β- and γ-secretases are supported by the distinct responses of control neuronal precursors to β-secretase and γ-secretase inhibition. Importantly, *bace1* knockout mice show increased proliferation but decreased neurogenesis in the adult brain (Chatila et al., 2018), whereas *psen1* knockout mice show increased neuronal differentiation (Handler et al., 2000). As single-cell expression datasets encompassing human aging and fAD hippocampi become available, it will be important to validate these findings.

It is intriguing that we see deficits in embryonic neuronal development, modeled by iPSC differentiation, whereas fAD clinical symptoms manifest from the fourth decade of life. Despite this disparity, evidence exists to suggest that fAD mutation carriers may have altered brain development. MRI scans have shown increased brain volume in children harboring *PSEN1* E280A mutations (Quiroz et al., 2015) and structural changes have been described in children who are *APOE4* carriers (Dean et al., 2014). However, these changes could reflect neuroinflammatory responses to early disease processes, in contrast to developmental effects. Despite these changes, our fAD iPSCs generate the appropriate proportions of cortical-layer markers after 100 days of differentiation (Arber et al., 2020), suggesting that neurogenic timing is altered, rather than developmental specification.

Studies suggest that hippocampal neurogenesis is reduced in the brains of patients with sporadic AD and mild cognitive impairment compared with age-matched controls (Moreno-Jiménez et al., 2019; Tobin et al., 2019). However, this remains controversial (Boldrini et al., 2018; Kempermann et al., 2018; Paredes et al., 2018; Sorrells et al., 2018; Tartt et al., 2018), and previous studies comparing AD postmortem brains have described opposing effects of the disease on neurogenesis (Boekhoorn et al., 2006; Crews et al., 2010; Jin et al., 2004). Our data describe neurogenesis in genetically confirmed fAD. The data do not support significant changes in adult hippocampal neurogenesis in fAD, yet they suggest some trends toward (1) reduced neurogenesis in fAD, supporting previous work on early-onset AD (Crews et al., 2010); (2) a disease-associated reduction in neurogenesis, despite younger NSC niches; and (3) mutation-specific effects, such as the reduced impact of the *PSEN1* M139V mutation on Notch cleavage compared with other mutations. These fAD cases show similar Braak staging. It will be interesting to further explore the relative contribution of tau and Aβ pathologies versus mutation-specific Notch signaling defects in neurogenesis dysfunction.

*In vivo* analysis of neurogenesis in fAD tissue is limited by the paucity of available tissue because of the rarity of the disease. In addition, we are unable to control for neurodegeneration, meaning that we cannot discount that cell death may have a more general impact on adult hippocampal neurogenesis. Indeed, neurogenesis and neuronal cell death have been shown to be interdependent and closely related to cognitive decline (Choi et al., 2018). Further experiments are required to test the hypothesis that fAD leads to a depletion of the stem cell pool with advancing age and contrast with the vulnerability of newborn neurons in relation to neurodegenerative processes. Defects in early-adult neurogenesis appear to be central for neuron vulnerability associated with later-stage AD in mouse models (Choi et al., 2018). This is reinforced as mice with a conditional *Psen1* knockout on a *Psen2* knockout background show stage-dependent effects on neurogenesis, with early enhancement of neurogenesis followed by a decline at late stages of disease with concomitant neuroblast vulnerability (Chen et al., 2008; Unger et al., 2016). The NESTIN staining presented here does not support an NSC depletion hypothesis but is in line with previous reports of unchanged NESTIN staining in AD model mice (Unger et al., 2016). Further analysis with alternative NSC- and Notch-associated immunohistochemistry is required to explore this question.

In summary, we have described a premature differentiation phenotype in human stem cell neurogenesis *in vitro* resulting from fAD mutations in *PSEN1*, with relevance to *in vivo* neurogenesis. *PSEN1* and *APP* mutations have distinct effects on neurogenesis, whereby our data support Notch signaling deficits that depend on *PSEN1* mutations, whereas overall neurogenesis is enhanced in organoids bearing *APP* mutations. These findings suggest that fAD mutations alter the timing of neurogenesis during development and throughout life, accelerating aging and predisposing neurodegeneration. These findings and genetic distinctions should be considered when future clinical studies targeting β- and γ-secretase are designed and analyzed.

## STAR★Methods

### Key Resources Table

**Table:** 

REAGENT or RESOURCE	SOURCE	IDENTIFIER
**Antibodies**		

b-III-tubulin	Biolegend	Cat#801201; RRID: AB_2313773
b-III-tubulin	Biolegend	Cat#802001; RRID: AB_2564645
Ki67	BD Bioscience	Cat#550609; RRID: AB_393778
APP C-terminal fragment	Biolegend	Cat#802803; RRID: AB_2715853
Aβ (6E10)	Biolegend	Cat#803014; RRID: AB_2728527
PSEN1	Millipore	Cat#MAB5232; RRID: AB_95175
Notch ICD (Val 1744)	Cell Signaling Technologies	Cat#4147; RRID: AB_2153348
β Actin	Sigma	Cat#1978; RRID: AB_476692
FOXG1	Abcam	Cat#ab18259; RRID: AB_732415
NESTIN	Santa Cruz	Cat#sc-23927; RRID:AB_627994
PAX6	Biolegend	Cat#901301; RRID:AB_2565003

**Chemicals, Peptides, and Recombinant Proteins**		

Dispase	ThermoFisher	17105-041
Accutase	ThermoFisher	A11105-01
SB431542	R&D Systems	1614/10
Dorsomorphin	R&D Systems	3093/10
N2 Supplement	ThermoFisher	17502-048
B27 Supplement	ThermoFisher	17504-044
POWER Sybr green	ThermoFisher	4368702
Trizol	ThermoFisher	15596-026
Essential 8 medium	ThermoFisher	A1517001
Laminin	Sigma	L2020
Matrigel	Corning	356255
DAPT (γ-secretase inhibitor N-[N-(3, 5-Difluorophenacetyl)-L-alanyl]-S-phenylglycine t-butyl ester)	Tocris	2634
LY2886721	Cambridge Bioscience	2299-5
Superscript IV	ThermoFisher	18091050
Geltrex	ThermoFisher	A1413302

**Critical Commercial Assays**		

Human (6E10) Aβ42 Ultra-Sensitive Kit	MesoScale Discovery	K151FUE

**Experimental Models: Cell Lines**		

Ctrl 1 - Human iPSC	Dr Tilo Kunath; Arber et al., 2020	N/A
Ctrl 2 - Human iPSC	Coriel cell repository	ND41886
Ctrl 3 - Human iPSC	Sigma	RBi001-a
Ctrl 4 - Human iPSC	Sigma	SIGi1001-a-1
Shef6 - Human ESC	UK Stem Cell Bank	N/A
APP V717I patient 1 (2 clones) - Human iPSC	Stembancc	N/A
APP V717I patient 2 - Human iPSC	Arber et al., 2020	N/A
PSEN1 int4del (2 clones) - Human iPSC	Stembancc	N/A
PSEN1 Y115H - Human iPSC	Arber et al., 2020	N/A
PSEN1 M139V - Human iPSC	Stembancc	N/A
PSEN1 M146I - Human iPSC	Stembancc	N/A
PSEN1 R278I (2 clones) - Human iPSC	Arber et al., 2020	N/A

**Oligonucleotides**		

*RPL18a* Fwd	CCCACAACATGTACCGGGAA	Arber et al., 2020
*RPL18a* Rev	TCTTGGAGTCGTGGAACTGC	Arber et al., 2020
*FABP7* Fwd	CAAGAACACGGAGATTAG	This study
*FABP7* Rev	GCTAACAACAGACTTACA	This study
*TUBB3* Fwd	CATGGACAGTGTCCGCTCAG	Arber et al., 2020
*TUBB3* Rev	CAGGCAGTCGCAGTTTTCAC	Arber et al., 2020

**Other**		

Expression data from human fetal tissue aged 15 – 21 weeks post conception	Miller et al., 2014	Miller et al., 2014
Single Cell RNaseq “dataset A” - dentate gyri from P12-P35 mice	Hochgerner et al., 2018	GEO: GSE95315

### Resource Availability

#### Lead Contact

Further information and requests for resources and reagents should be directed to and will be fulfilled by the Lead Contact, Charlie Arber c.arber@ucl.ac.uk).

#### Materials Availability

This study did not generate new unique reagents.

#### Data and Code Availability

This study did not generate any unique datasets or code. Published datasets analyzed in this study are GEO: GSE95315 (Hochgerner et al., 2018), and http://brainspan.org/ (Miller et al., 2014). Sequencing analysis code can be found at (https://github.com/harrislachlan/data).

### Experimental Model and Subject Details

#### Details of stem cell lines employed

**Table:** 

Cell line	No. of clones	Name and origin	Sex	Age at onset	Age at biopsy	APOE status
Ctrl1		Dr Tilo Kunath	M		78	3/3
Ctrl2		ND41886 Coriel	M		64	2/3
Ctrl3		RBi001-a Sigma	M		45-49	3/3
Ctrl4		SIGi1001-a-1 Sigma	F		20-24	3/4
Shef6 (hESC)	1	UK Stem Cell Bank	F		n/a	3/3
*APP* V717I	2	StemBancc	M	49	58	4/4
*APP* V717I-2	1	Arber et al., 2020	F	Asymptomatic	47	3/3
*PSEN1* int4del	2	Stembancc	F	47	47	3/3
*PSEN1* Y115H	1	Arber et al., 2020	M	34	39	3/3
*PSEN1* M139V	1	Stembancc	F	34	45	2/3
*PSEN1* M146I	1	Stembancc	M	Asymptomatic	38	3/3
*PSEN1* R278I	2	Arber et al., 2020	M	58	60	2/4

#### Case information for post-mortem brain tissue

Fresh frozen post-mortem hippocampal tissue was made available through the Queen Square Brain Bank for Neurological Disorders. Tissue from five controls and seven fAD cases were available. Experimental group allocation was made via gene mutation status.

**Table:** 

	**PM delay**	**AAO**	**AAD**	**Duration**	**Gender**	**Clinical Diag**	**Path Diag**	**Brain Weight**	**Mutations**	**APOE**	**Braak Tau**	**Thal Phase**	**CERAD**	**ABC**	**CAA**
Control	16:15:00	na	88	na	M	Control	Path Aging	1077	Control 1	33	2		2	B1	0
Control	76:10:00	na	71	na	F	Control	low level AD changes	1214	Control 2	33	3	2	0	A1B2C0	1
Control	47:05:00	na	89	na	M	Control	Control	1356	Control 3	33	2	3	1	A2B1C1	1
Control	59:57:00	na	96	na	F	Control	Control	1032	Control 4	33	2	2	0	A1B1C0	1
Control	38:00:00	na	96	na	F	Control	Control	1092	Control 5		2	1	0	A1B1C0	1
fAD	32:10:00	49	62	13	M	fAD	fAD	1302	APP V717L	44	6	5	3	A3B3C3	3
fAD	68:05:00	60	66	6	M	AD	fAD	1437	APP V717I	33	6	5	3	A3B3C3	1
fAD	43:10:00	42	51	9	M	fAD	fAD	1415	PSEN1 int4del	33	6	5	3	A3B3C3	3
fAD	128:35:00	41	49	8	F	fAD	fAD	1205	PSEN1 M139V	33	6	5	3	A3B3C3	2
fAD	77:45:00	51	66	15	M	fAD	fAD	1106	PSEN1 R278I	23	6	5	3	A3B3C3	3
fAD	11:00:00	42	53	11	F	fAD	fAD	994	PSEN1 E280G	34	6	5	3	A3B3C3	3
fAD	43:50:00	42	47	5	M	MSA	fAD	1225	PSEN1 A434T & T291A	33	5	5	3	A3B3C3	3

Abbreviations; fAD – familial Alzheimer’s disease, PM delay -post mortem delay, AAO – age at onset, AAD – age at death, CAA – cerebral amyloid angiopathy score

### Method Details

#### Cell culture

The iPSC lines employed in the study have been described previously (see STAR Methods; Arber et al., 2019, 2020). Where possible, data was corroborated with two iPSC clones from an individual donor, however, focus was made on increasing the number of individual donors to mitigate genetic variation and reduce false results (Germain and Testa, 2017). iPSCs were maintained in Essential 8 media on geltrex substrate and mechanically passaged using 0.5mM EDTA (all reagents ThermoFisher unless stated). Differentiation was employed following published protocols (Shi et al., 2012). Briefly, neural commitment was initiated with 10 μM SB431542 and 1 μM dorsomorphin (both R&D). Further patterning was performed in N2B27 media with retinoic acid. Cells were plated at day 18 on laminin (Sigma) coated 96 well plates and imaged at day 27.

Cerebral organoids were generated following published protocols (Lancaster et al., 2013). 9,000 iPSCs were seeded into embryoid bodies (EBs). EBs were embedded into Matrigel (Corning) on day 8 and maintained until day 45 at which point they were fixed in 4% para-formaldehyde for 2 hours, flushed with 30% sucrose solution and embedded in OCT cryo-embedding matrix. 10 μm sections were taken on a cryostat (Leica CM 1520).

Treatments were as follows; DAPT (N-[N-(3, 5-Difluorophenacetyl)-L-alanyl]-S-phenylglycine t-butyl ester, Tocris) at 10 μM and LY2886721 (Cambridge Bioscience) at 1 μM, both for 5 days. Fold changes were measured relative to DMSO vehicle controls within each treatment plate.

#### Aβ42 ELISAs

48 hour conditioned media was collected and centrifuged at 2,000 g to remove cell debris. The human (6E10) Aβ42 ultra-sensitive electrochemiluminescent kit (Meso Scale Discovery) was used as per the manufacturers instructions.

#### Immunocytochemistry

Cells were fixed in 4% paraformaldehyde for 15 minutes, and subsequently permeabilised with 3 washes of PBST supplemented with 0.3% Triton X-100 (PBST). Blocking was then performed with 3% bovine serum albumin (Sigma) in PBST for 20 minutes at room temperature. Primary antibodies (listed in the Key Resources Table) were incubated in blocking solution overnight followed by three washes in PBST. Secondary antibodies (Alexafluor, Thermo) were incubated for 1 hour in the dark in blocking solution. DAPI nuclear counterstain was added in one of the final three PBS washes. Immunocytochemistry was performed in the same way on organoid sections.

Organoid relative progenitor-neural contribution was quantified at day 45. At least two neurogenic regions per organoid were selected based on morphology and FOXG1/PAX6/TUJ1 immunocytochemistry (for example, see Figure 1B). Within neurogenic regions, measurements were performed from basal membrane to neural boundaries in μm as depicted in Figure 4A. Measurements from basal membranes ensured full transverse sections of the neurogenic niche. Image capture and quantification was done in a genotype blinded manner.

High content imaging was performed on the Perkin Elmer Opera Phenix. At least 1000 cells were imaged per well in duplicate per time point and analysis was performed on the Columbus software. Images of organoid sections were taken on a Zeiss LSM microscope and analyzed using Leica LAS software. No post hoc manipulation was performed. Neurogenic scoring was performed blinded on the Leica LAS software.

#### Immunohistochemistry

10 μm sections of fresh frozen hippocampus were taken on the cryostat (Leica CM 1520) and stored at −80 degrees. Sections were fixed in 4% paraformaldehyde for 15 minutes. Staining was performed as above, however, secondary biotinylated antibodies and ABC signal amplification were used following primary antibody incubation. Signal was developed using DAB staining and counterstained with Meyer’s solution before dehydration, scoring and imaging. Quantification was made for each marker per 200 μm planar length of dentate gyrus. Scoring was performed in a genotype-blinded manner by three independent scorers. All case information is listed in STAR Methods.

#### qPCR

RNA was isolated using Trizol solution as per the manufacturers protocol. 2 μg of total RNA was reverse transcribed using SuperScript IV reverse transcriptase with random hexamer primers. qPCR was performed on an Agilent MX3000P with Power Sybr Green master mix (primer sequences in Key Resources Table). Analysis was performed using the ΔΔCT method.

#### Western blotting

Cells were lysed in RIPA buffer and 15-25 μg of total protein, determined using BCA assay (Biorad), was loaded onto precast gels Bis-Tris polyacrylamide gels (NuPage). After running in MES buffer, proteins were transferred onto a nitrocellulose membrane and blocked in 3% bovine serum albumin. Primary antibodies were incubated overnight in blocking solution. After incubation with secondary antibodies (Rockland), imaging and analysis were performed on the Li-Cor Odyssey Fc.

#### BrainSpan data

Expression data from human fetal tissue aged 15 – 21 weeks post conception was leveraged from http://brainspan.org/ (Miller et al., 2014). Z scores were calculated across the developmental cortical layers and data was presented as a heatmap in Figure 1.

#### Analysis of single cell RNA-seq data

The mapped single-cell RNA sequencing dataset A (GSE95315) from the originating study (Hochgerner et al., 2018) were read into Seurat (Ver. 3.1.2) for analysis (Stuart et al., 2019). The dataset A contains the transcriptomes of 5, 454 cells from mouse dentate gyri ranging from P12-P35. The adult hippocampal NSCs (AH-NSCs), intermediate neuronal progenitor cells (IPCs) and granule neuron clusters were identified according to marker gene expression. Briefly, the cluster of AH-NSCs was identified based on a *Apoe-high/Hopx*-high/*Aqp4-low* expression profile (Harris et al., 2020), whereas IPCs had high expression of *Eomes* and of a variety of cell-cycle marker genes (e.g *Mcm2-7*, *Mki67*). Finally, the granule neuron cluster was identified based on high expression of *Tubb3*, *Prox1* and the absence of the aforementioned markers. Differential gene expression testing between AH-NSCs and granule neurons was performed using the FindMarkers function in Seurat. This function determines differential expression by comparing gene expression levels across all cells in a particular cluster (not just positive cells). The tests were performed on the normalized data located in the “data” slot of the “RNA” assay (Hafemeister and Satija, 2019). Genes were tested for differential gene expression if they were expressed by at least 10% of cells in at least one of the cellular clusters being compared. No minimum log-fold change threshold was enforced. Statistical significance (p < 0.05) was determined using Wilcoxon rank sum tests and the P*-*value was corrected for multiple-testing using the False Discovery Rate. All analysis was performed in R (3.6.2).

### Quantification and Statistical Analysis

#### Statistics

Data were collated in Microsoft Excel and GraphPad Prism 7. Normality was tested via D’Agostino-Pearson, or Shapiro-Wilk tests in the case of fewer repeats. Pooled controls and pooled mutation lines were compared via ANOVA with post hoc Tukey as indicated in the figure legends. See Table S1 for all tests used, exact P values, confidence intervals and degrees of freedom. P values are represented by ^∗^ p < 0.05, ^∗∗^ p < 0.01, ^∗∗∗^ p < 0.001. Multiple independent neural inductions were performed for each iPSC line and exact numbers are shown within histograms.

## Supplemental Information

Document S1. Figures S1–S4

Table S1. Details of Statistical Analyses, Related to Figures 2, 3, 4, and 6

Document S2. Article plus Supplemental Information
