# Supplementary material for: Familial Alzheimer’s Disease Mutations in PSEN1 Lead to Premature Human Stem Cell Neurogenesis
Source: Cell Rep. 2021 Jan 12;34(2):108615. doi: 10.1016/j.celrep.2020.108615 (PMC7809623; doi:10.1016/j.celrep.2020.108615)
Supplement: Document S1. Figures S1–S4 [file mmc1.pdf]

Cell Reports, Volume *34*

Supplemental Information

Familial Alzheimer's Disease Mutations in *PSEN1* Lead to Premature Human Stem Cell Neurogenesis

Charles Arber, Christopher Lovejoy, Lachlan Harris, Nanet Willumsen, Argyro Alatza, Jackie M. Casey, Georgie Lines, Caoimhe Kerins, Anika K. Mueller, Henrik Zetterberg, John Hardy, Natalie S. Ryan, Nick C. Fox, Tammaryn Lashley, and Selina Wray

Figure S1

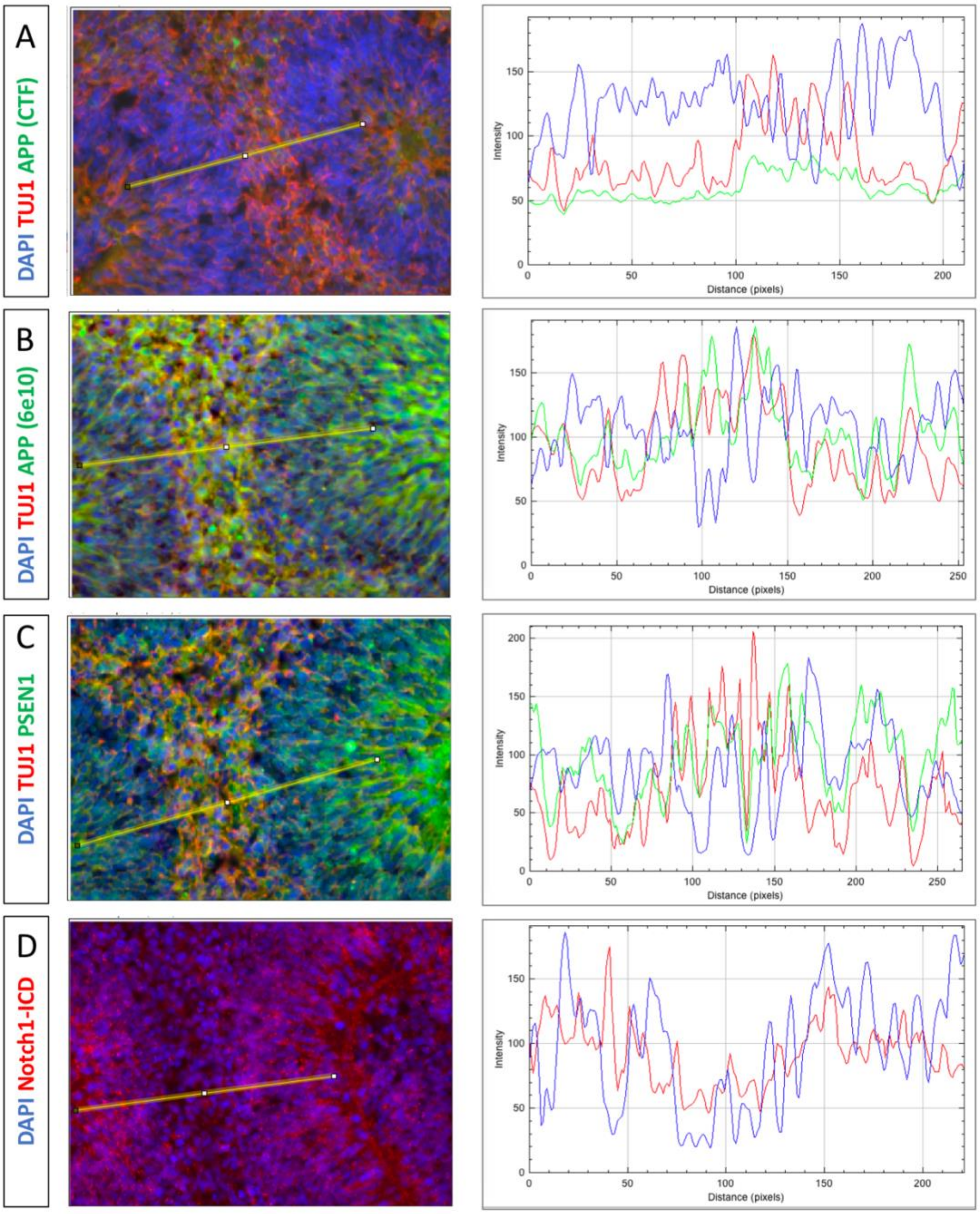

Fig S1 – Intensity analysis of images in Fig 1B.
A-B) APP immunostaining (C terminal fragment and 6E10 recognising Aβ) shows increased intensity in the neural regions (marked by TUJ1). C) PSEN1 immunostaining shows a more homogeneous expression across pro-neural and neural regions. D) Notch intracellular domain (Notch ICD) is enriched in neural progenitor regions. A-D are adjacent sections.

Figure S2

A

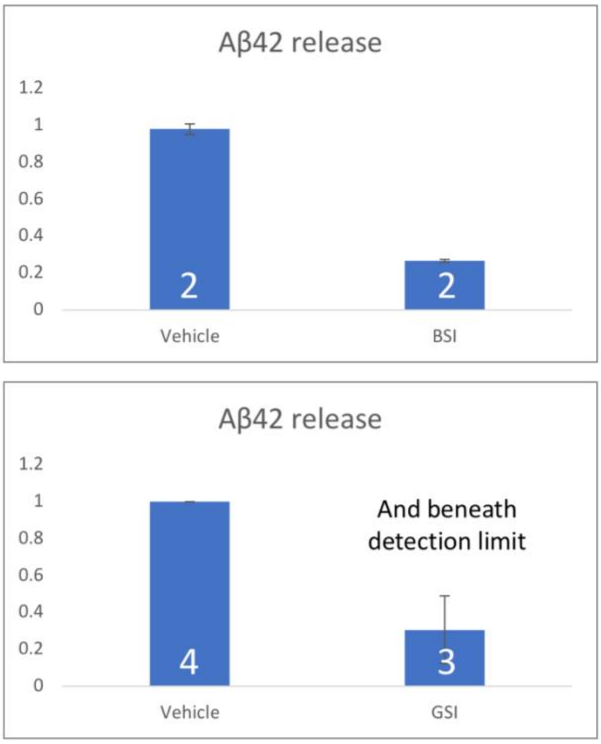

B

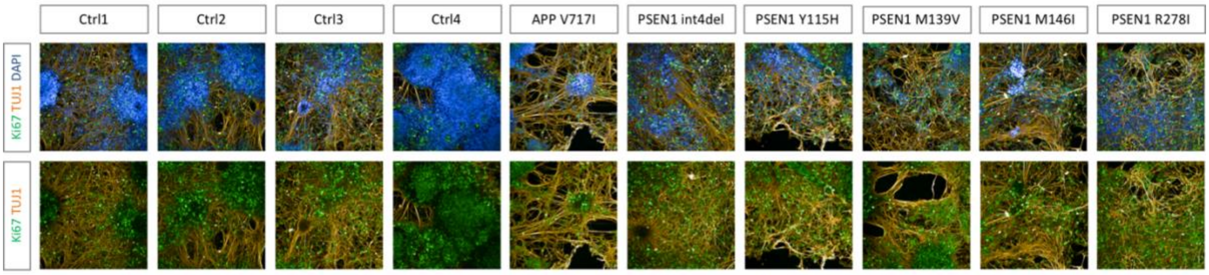

Figure S2 – Aβ42 ELISA and images of all iPSC lines at 27 DIV.

A) ELISA measurement of Aβ42 release from neuronal cultures to confirm the action of β- and γ-secretase inhibitors (see Fig 2). B) Representative images of all lines at day 27 of induction, used for quantification of Ki67 proliferating progenitors and TUJ1 post-mitotic neurons (see Fig 3).

Figure S3

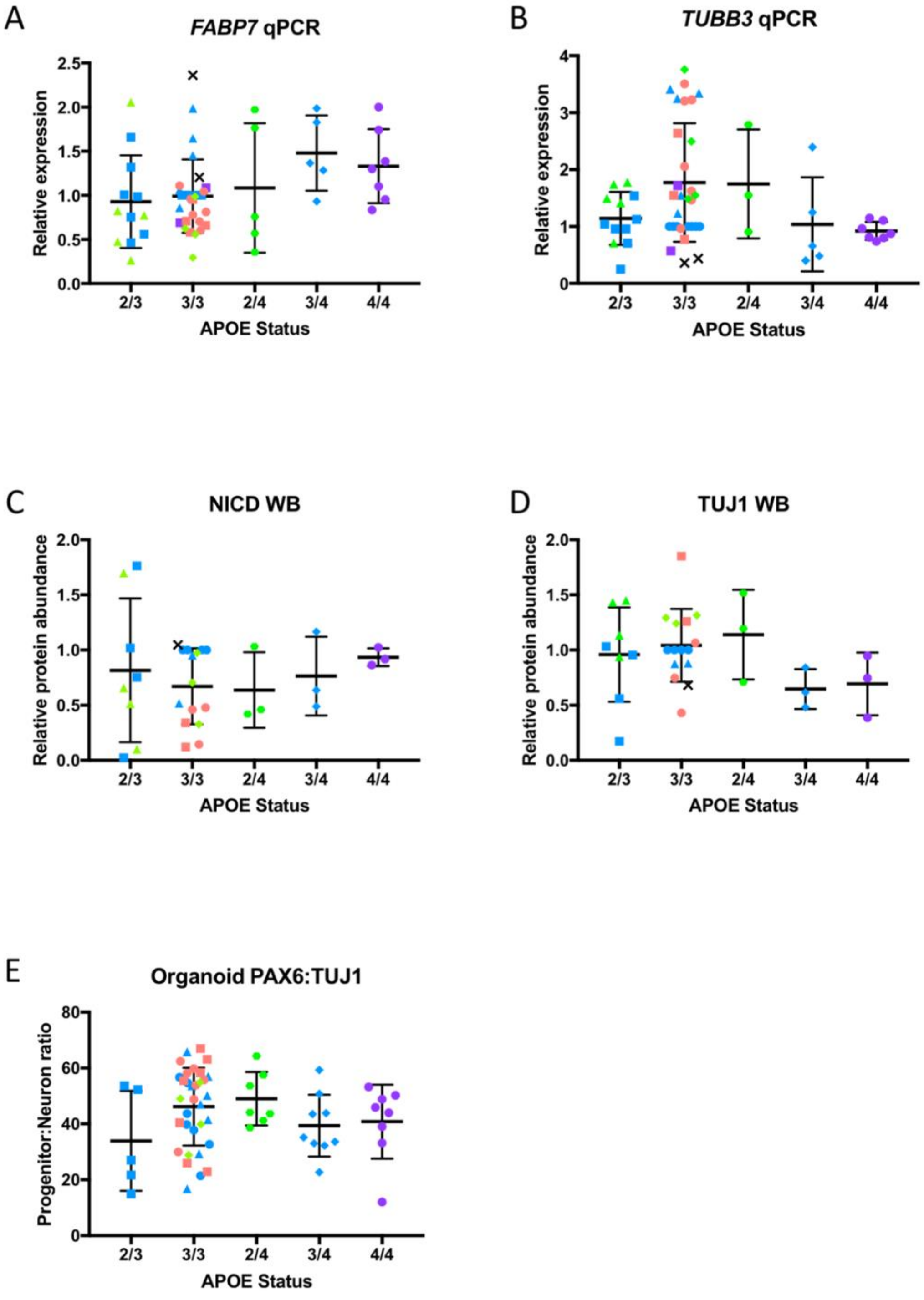

Figure S3 – Data from Fig 3 and Fig 4 regrouped via APOE genotype.
Symbols: Ctrl1 – blue circles. Ctrl2 – blue squares. Ctrl3 – blue triangles. Ctrl4 – blue diamonds. hESC – black crosses. *APP* V717I – purple circles. *PSEN1* int4del – pink circles. *PSEN1* Y115H – pink squares. *PSEN1* M139V – green triangles. *PSEN1* M146I – green diamonds. *PSEN1* R278I – green hexagons.

Figure S4

A

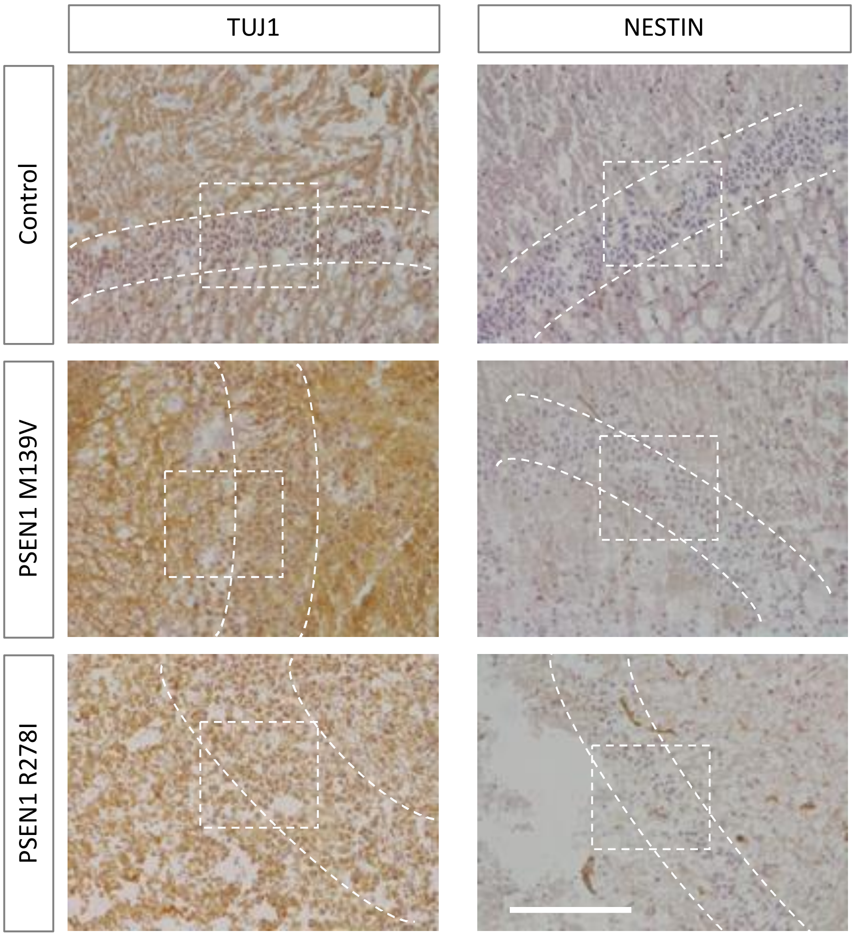

B

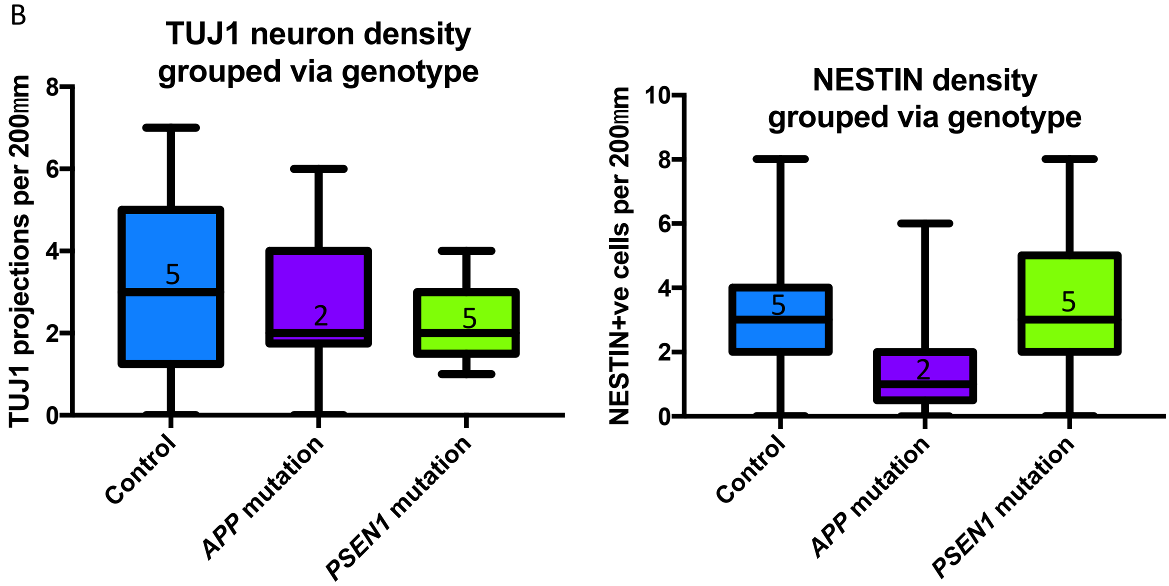

Figure S4 – Low magnification images of post mortem hippocampi and quantification grouped via genotype. A) Low magnification images for Fig 6. Boxes represent images from Fig 6 and dotted lines depict the granule layer of the dentate gyrus. Scale bar represents 200µm. B) Analysis for TUJ1 neurite density and NESTIN staining in hippocampi grouped via genotype.
