# Supplementary material for: Familial Alzheimer’s Disease Mutations in PSEN1 Lead to Premature Human Stem Cell Neurogenesis
Source: Cell Rep. 2021 Jan 12;34(2):108615. doi: 10.1016/j.celrep.2020.108615 (PMC7809623; doi:10.1016/j.celrep.2020.108615)
Supplement: Table S1. Details of Statistical Analyses, Related to Figures 2, 3, 4, and 6 [file mmc2.docx]

Table S1. Details of statistical analyses related to Figures 2, 3, 4 and 6.

|  |  |  | Test | P value |  | DoF | 95% CI |
| --- | --- | --- | --- | --- | --- | --- | --- |
|  |  |  |  |  |  |  |  |
| Figure 2 | B nuclei | BSI v DMSO v GSI | ANOVA post hoc Tukey | ns post hoc | * |  |  |
|  |  | BSI v DMSO | ANOVA post hoc Tukey |  | ns | 15 |  |
|  |  | DMSO v GSI | ANOVA post hoc Tukey |  | ns | 15 |  |
|  | C Ki67 | BSI v DMSO | ANOVA post hoc Tukey |  | ns | 15 | -0.3462 to 0.5128 |
|  |  | BSI v GSI | ANOVA post hoc Tukey | 0.0092 | ** | 15 | 0.1425 to 1.001 |
|  |  | DMSO v GSI | ANOVA post hoc Tukey | 0.0251 | * | 15 | 0.05918 to 0.9182 |
|  | D TUJ1 | BSI v DMSO | ANOVA post hoc Tukey |  | ns | 15 | -1.369 to 1.586 |
|  |  | BSI v GSI | ANOVA post hoc Tukey | 0.0137 | * | 15 | -3.334 to -0.3784 |
|  |  | DMSO v GSI | ANOVA post hoc Tukey | 0.0093 | ** | 15 | -3.442 to -0.4871 |
|  |  |  |  |  |  |  |  |
|  |  |  |  |  |  |  |  |
|  |  |  |  |  |  |  |  |
| Figure 3 | B Ki67 | Pooled Ctrl v PSEN1 int4del and Y115H | ANOVA post hoc Tukey | 0.0081 | ** | 52 | 6.795 to 52.91 |
|  |  | Pooled Ctrl v Pooled PSEN1 |  |  | ns |  |  |
|  | B TUJ | Pooled Ctrl v Pooled PSEN1 | ANOVA post hoc Tukey |  | ns |  |  |
|  |  |  |  |  | ns |  |  |
|  | D NICD | Pooled Ctrl v Pooled PSEN1 | ANOVA post hoc Tukey |  | ns |  |  |
|  |  | Pooled Ctrl v PSEN1 int4del and Y115H | ANOVA post hoc Tukey | 0.0047 | ** | 35 | 0.1176 to 0.7186 |
|  | D PAX6 | Pooled Ctrl v Pooled PSEN1 | ANOVA post hoc Tukey | ns |  |  |  |
|  | D TUJ1 | Pooled Ctrl v Pooled PSEN1 | ANOVA post hoc Tukey | 0.0028 | ** | 49 | -0.5014 to -0.09243 |
|  |  | Pooled Ctrl v PSEN1 int4del and Y115H | ANOVA post hoc Tukey |  | ns |  |  |
|  |  |  |  |  |  |  |  |
|  | E FABP7 | Pooled Ctrl v Pooled PSEN1 | ANOVA post hoc Tukey | 0.0083 | ** | 54 | 0.09249 to 0.7302 |
|  |  | Pooled Ctrl v PSEN1 int4del and Y115H | ANOVA post hoc Tukey | 0.0112 | * | 40 | 0.09013 to 0.8078 |
|  | E TUBB3 | Pooled Ctrl v Pooled PSEN1 | ANOVA post hoc Tukey | 0.0310 | * | 57 | -1.345 to -0.04709 |
|  |  | Pooled Ctrl v PSEN1 int4del and Y115H | ANOVA post hoc Tukey | 0.0457 | * | 45 | -1.702 to -0.01189 |
|  |  |  |  |  |  |  |  |
| Figure 4 | C | Pooled Ctrl v Pooled PSEN1 | ANOVA post hoc Tukey | 0.0266 | * | 63 | 0.9231 to 18.15 |
|  |  |  |  |  |  |  |  |
|  | D | Pooled Ctrl v Pooled APP | ANOVA post hoc Tukey | 0.0133 | * | 63 | -108.6 to -10.58 |
|  |  |  |  |  |  |  |  |
| Figure 6 |  |  |  |  |  |  |  |
|  | B M139V | M139V v other PSEN1 cases pooled | ANOVA post hoc Tukey | 0.0023 | ** | 156 | 0.5059 to 4.33 |
|  |  |  |  |  |  |  |  |
|  | E AAD | All data | R-sqaured | 0.03685 |  |  |  |
|  |  | Control | R-sqaured | 0.0233 |  |  |  |
|  |  | PSEN1 | R-sqaured | 0.00095 |  |  |  |
|  |  |  |  |  |  |  |  |
|  | F DD | All data | R-sqaured | 0.0491 |  |  |  |
|  |  | PSEN1 | R-sqaured | 0.0006374 |  |  |  |

Abbreviations; DoF – degrees of freedom, BSI – β-secretase inhibitor, GSI - γ-secretase inhibitor, CI – confidence interval, NICD – Notch intracellular domain.
